# How *Shigella* tackles host defences

**DOI:** 10.7554/eLife.109371

**Published:** 2025-11-03

**Authors:** Yizhou Huang, Teresa LM Thurston

**Affiliations:** 1 Sir William Dunn School of Pathology, University of Oxford Oxford United Kingdom

**Keywords:** bacteriology, Shigella, ubiquitin, RNF213, xenophagy, autophagy, Other

## Abstract

The pathogenic bacteria *Shigella* avoids detection inside hosts cells by degrading RNF213, the protein responsible for sensing the presence of intracellular pathogens.

**Related research article** Saavedra-Sanchez L, Dickinson MS, Apte S, Zhang Y, de Jong M, Skavicus S, Heaton NS, Alto NM, Coers J. 2024. The *Shigella flexneri* effector IpaH1.4 facilitates RNF213 degradation and protects cytosolic bacteria against interferon-induced ubiquitylation. *eLife* **13**:RP102714. doi: 10.7554/eLife.102714.

Despite being single-celled organisms that are invisible to the naked eye, bacteria have profoundly shaped the world we live in, and they continue to do so. Bacteria had an essential role in the evolution of complex cells, are the basis of the food web, and are often good for our health. However, a small number of pathogenic bacteria can cause devasting diseases, and the evolution of anti-microbial resistance is increasing the threat presented by these bacteria.

The immune response protects us against bacteria and other pathogens. A variety of receptors survey the environment, both inside and outside cells, for foreign material – and when such material is detected, the immune system releases a variety of cells to neutralize the threat. Recently researchers discovered a receptor called RNF213 (short for ring finger protein 213) that is able to detect an unusually wide range of pathogens when they enter our cells, including bacteria, viruses and various parasites (Crespillo-Casado et al., 2024; Otten et al., 2021; Thery et al., 2021). This raises an obvious question: how do the intracellular pathogens that encounter RNF213 overcome its protective function to establish an infection?

Now, in eLife, Jörn Coers and colleagues at Duke University Medical Center – including Luz Saavedra-Sanchez as first author – report the results of experiments which show how a bacterial pathogen called *Shigella* is able to inhibit the function of RNF213 (Saavedra-Sanchez et al., 2024). The various species of *Shigella* cause dysentery and diarrhea, killing hundreds of thousands of people every year. Central to the pathogenicity of *Shigella* is a specialised nanomachine that injects virulence factors into the host cell. These factors disrupt and change the normal functions of the host, creating an environment that is beneficial to the bacteria.

Saavedra-Sanchez et al. infected human cells with *Shigella* and monitored their ability to mediate an immune response. Specifically, the researchers concentrated on whether the bacteria became surrounded by ubiquitin – a small, highly-conserved, regulatory protein and signalling molecule that RNF213 uses to “mark” invading pathogens. They found that wild-type *Shigella* were rarely marked with ubiquitin. However, a mutant form of the bacteria – a form that was unable to deliver virulence factors – was almost always marked with ubiquitin, courtesy of RNF213 (Figure 1A). Further experiments revealed that a particular virulence factor – an effector protein called IpaH1.4 – had a critical role in the suppression of RNF213-mediated ubiquitin tagging (Figure 1B). Moreover, and in line with other recent publications analysing how *Shigella* avoids RNF213 activity (Naydenova et al., 2025; Zhou et al., 2025), they showed that IpaH1.4 can induce the destruction of RNF213 by – somewhat ironically – marking it with ubiquitin. Degrading host proteins, via the addition of ubiquitin, has become a common signature of how *Shigella* uses IpaH proteins to block the host immune response.

**Figure 1.**
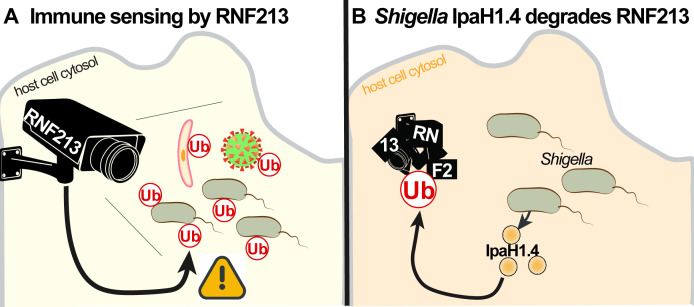
Shigella versus RNF213. (**A**) RNF213 (ring finger protein 213) is an immune sensor that can detect a wide range of intracellular pathogens, including various bacteria (grey), viruses (green), and parasites (yellow). It marks these pathogens with ubiquitin (Ub), and they are subsequently destroyed by the host cell. (**B**) The intracellular bacteria *Shigella* escapes the attention of RNF213 by secreting a virulence factor called IpaH1.4 (orange circles), which tags RNF213 with ubiquitin, thus preventing RNF213 from marking the bacteria with ubiquitin.

Saavedra-Sanchez et al. then went on to make a curious observation while studying the ubiquitin chains that surround the *Shigella* mutants. A particular type of chain called M1-linked ubiquitin (in which the ubiquitin molecules are linked head-to-tail) was forming, even though an enzyme called LUBAC – which is the only enzyme known to generate chains of this type – was not present. This meant that a different enzyme, which might have been RNF213 itself, was responsible for marking the bacteria with M1-linked ubiquitin. Interestingly, Coers and colleagues have observed M1-linked ubiquitin chains being formed in the absence of LUBAC before: in these experiments the chains formed on a different bacterial pathogen, *Chlamydia trachomatis* (Walsh et al., 2022). As M1-linked ubiquitin regulates numerous pathways within our cells, if this LUBAC-independent pathway is widespread, this finding could have a profound impact on biology.

Finally, how different pathogens overcome our host immune defences has been a long-standing area of research. It is thus interesting to note that *Shigella* is not the only pathogen to target RNF213. Other intracellular bacteria, including *Burkholderia* and *Chlamydia*, also target it (Szczesna et al., 2024; Walsh et al., 2022). Therefore, whilst the mechanisms of RNF213 inhibition diverge, evolution is telling us that this sensor is important to how the immune system responds to diverse intracellular pathogens. Whether RNF213 represents a potential target for novel therapeutic approaches in the treatment of bacterial infections will be an interesting area of future research.
